# Therapeutic communication and its associated factors among nurses working in public hospitals of Gamo zone, southern Ethiopia: application of Hildegard Peplau’s nursing theory of interpersonal relations

**DOI:** 10.1186/s12912-023-01526-z

**Published:** 2023-10-13

**Authors:** Abera Mersha, Abebe Abera, Temamen Tesfaye, Tesfaye Abera, Admasu Belay, Tsegaye Melaku, Misaye Shiferaw, Shitaye Shibiru, Wubshet Estifanos, Senahara Korsa Wake

**Affiliations:** 1 School of Nursing, College of Medicine and Health Sciences, Arba Minch University, Arba Minch, Arba Minch, Ethiopia; 2 School of Nursing, Institute of Health, Jimma University, Jimma, Ethiopia; 3 School of Nursing and Midwifery, Institute of Health Sciences, Wollega University, Nekemte, Ethiopia; 4 School of Pharmacy, Institute of Health, Jimma University, Jimma, Ethiopia; 5 Public Health Specialist, Addis Ababa City Administration Health Bureau, Addis Ababa, Ethiopia; 6 College of Natural and Computational Science, Ambo University, Ambo, Ethiopia

**Keywords:** Therapeutic communication, Nurse-patient relationship, Gamo zone

## Abstract

**Background:**

Therapeutic communication can assist nurses in achieving their goals. Effective nurse-patient communication can improve clinical outcomes and boosts patient satisfaction. But, there is an arming gap in therapeutic communication between nurses and patients in Ethiopia, which hinders the quality of nursing care. Some studies have been done on therapeutic and its barriers. Nevertheless, those studies did not fully address factors from different perspectives and were supported by nursing theories or models. Therefore, this study aimed to fill these gaps in the study setting.

**Methods:**

Institution-based cross-sectional study was conducted among 408 nurses working in public hospitals of Gamo zone from December 1, 2021, to January 30, 2022. Out of the six hospitals in the Gamo zone, three were selected by simple random sampling method. The data were collected by an interview-administered Open Data Kit survey tool and analyzed by SAS version 9.4. Descriptive statistics were computed and a generalized linear model was used to identify associated factors.

**Results:**

In this study, a standardized percentage of the maximum scale of therapeutic communication was 52.32%. Of the participants, 40.4% had high, 25.0% moderate, and 34.6% had low levels of therapeutic communication. Age, marital status, and qualification showed significant and positive relationships with the overall therapeutic communication. However, sex, working unit, nurse burnout, lack of empathy from nurses, challenging nursing tasks, lack of privacy, use of technical terms by nurses, lack of confidence in nurses, stress, unfamiliarity with the nursing job description, shortage of nurses, insufficient knowledge, lack of participation in decision making, and having contagious disease showed a significant and negative relationship with overall therapeutic communication.

**Conclusions:**

This finding indicates a gap in therapeutic communication between nurses and patients, and modifiable factors are identified. Therefore, giving opportunities for nurses to improve their qualifications, a special attention to nurses working in stressful areas, sharing the burden of nurses, involving nurses and patients in decision-making, and motivating and creating a positive working environment is vital to improving therapeutic communication.

**Supplementary Information:**

The online version contains supplementary material available at 10.1186/s12912-023-01526-z.

## Background

Therapeutic communication is the fundamental component of nursing that focuses on advancing physical, mental, and emotional well-being [1]. Nurses use therapeutic communication techniques to provide support and information to patients [2–4]. Nurses integrate diverse therapeutic communication techniques while communicating with client to accomplish their goals or objectives [3]. Those techniques are silence, accepting, giving recognition, offering self, giving broad openings, active listening, seeking clarification, placing the event in the sequence, making observations, encouraging descriptions of perception, encouraging comparisons, summarizing, reflecting, focusing, confronting, voicing doubt, and offering hope and humor [2, 5–8].

Meaningful relationships will allow nurses to carry out their clinical job easily by keeping patients engaged in their care. Forging meaningful nurse-patient relationships is accomplished by making small changes to the nursing workflow [5, 9]. Effective nurse-patient communication is a core component of nursing care, and it improves clinical outcomes and boosts patient satisfaction. When communication is a patient-centered and nurse-patient dyad, it becomes therapeutic and allows for trust and mutual respect, addressing patients’ and caregivers’ needs, concerns, and preferences. However, effective nurse-patient communication is the biggest challenge for nurses and requires much more experience and skills [10, 11]. Nurses need balance compassion, clinical expertise, and technology demands to create a quality care encounter that supports patient health and emotional needs [9].

Shreds of evidence from different studies conducted in India, Indonesia, Turkey, and Iran stated that effective nurse-patient therapeutic communication improves patient satisfaction with nursing care services provided in healthcare institutions [12–17]. However, ineffective communication contributes to poor outcomes, decreased quality of nursing care, medical errors, and psychological stress for patients. It can also cause them to feel unsafe and uninformed, perceived staff as inexperienced, incompetent, or unknowledgeable [6].

Some studies have been done on therapeutic communication and its barriers in different parts of the world, including Ethiopia. Nevertheless, those studies did not fully address factors from different perspectives and not supported by nursing theories or models. Majority of the studies were qualitative and done on a few samples, which may put generalizability issues under caution. Therefore, this study aimed to assess the status of therapeutic communication and its associated factors by applying Hildegard Peplau’s nursing theory of interpersonal relations.

## Methods

### Study setting, design, and period

This cross-sectional study was conducted in public hospitals of the Gamo zone, southern Ethiopia, from December 1, 2021, to January 30, 2022. Gamo zone is one of the administrative zones in southern Ethiopia. There are six hospitals (one general and five primary) in the Gamo zone. These are Arba Minch General Hospital, Dilfana Primary Hospital, Chencha Primary Hospital, Kamba Primary Hospital, Gerese Primary Hospital, and Selamber Primary Hospital. However, this study was conducted only at Arba Minch General Hospital, Kamba Primary Hospital, and Chencha Primary Hospital.

### Population

The source population for this study was all nurses working in public hospitals of Gamo zone, southern Ethiopia. Nevertheless, those working in the medical and surgical ward during the data collection period were the study population.

### Eligibility

All nurses who completed the probation period in the study hospitals were included in this study. However, those working less than a month in the respective ward, nurses who come for help, and those on annual leave were excluded.

### **Sample size determination and sampling procedure**

A Cochran’s formula was used to estimate the sample size in the OpenEpi version 3.01 online software. The assumptions were 61.4% of the proportion of the effective communication skills to the patient from a study conducted in Ethiopia [18], a 95%CI, a 5% margin of error, and a 10% non-response rate. Based on the stated assumptions, the calculated sample size for this study was 402. However, the estimated sample size was approximately equal to the number of nurses working in the respective wards of the study hospitals, which were 419. As such, all the nurses who met the inclusion criteria were involved in the study to increase the precision. Out of the six hospitals in the Gamo zone, three were selected by simple random sampling method.

### Data collection method

The data were collected by a structured interviewer-administered Open Data Kit (ODK) survey tool. The tool was adapted by reviewing different works of literature and contain six main parts: socio-demographic characteristics, nurse-related factors, patient-related factors, environmental factors, personal/social factors, and questions to assess therapeutic communication [1, 19–24]. There are a total of 28 questions to measure the six dimensions of therapeutic communication (Additional file 1). Seven data collectors who qualify for BSc degrees in nursing were involved, and two nurses with MSc degree qualifications were involved in supervision. The data were collected from nurses in medical and surgical wards of the hospitals during the study period. A two-days training was given for the data collectors and supervisors. The data were collected through a face-to-face interview in the hospital setting.

### Operational definitions and measurements

#### Therapeutic communication

A therapeutic relationship between nurse and patient containing three sub-factors such as, empathy, trust and rapport, and power-sharing based on Global Interprofessional Therapeutic Communication Scale (GITCS©) [23]. A standardized percentage of the maximum scale (%SM) of therapeutic communication was computed by using the following formula.

$$\varvec{\%}\varvec{S}\varvec{M}=\frac{\varvec{A}\varvec{c}\varvec{t}\varvec{u}\varvec{a}\varvec{l} \varvec{m}\varvec{e}\varvec{a}\varvec{n} \varvec{s}\varvec{c}\varvec{o}\varvec{r}\varvec{e}-\varvec{S}\varvec{c}\varvec{a}\varvec{l}\varvec{e} \varvec{m}\varvec{i}\varvec{n}\varvec{i}\varvec{m}\varvec{u}\varvec{m} \varvec{s}\varvec{c}\varvec{o}\varvec{r}\varvec{e}}{\varvec{S}\varvec{c}\varvec{a}\varvec{l}\varvec{e} \varvec{m}\varvec{a}\varvec{x}\varvec{i}\varvec{m}\varvec{u}\varvec{m} \varvec{s}\varvec{c}\varvec{o}\varvec{r}\varvec{e}-\varvec{S}\varvec{c}\varvec{a}\varvec{l}\varvec{e} \varvec{m}\varvec{i}\varvec{n}\varvec{i}\varvec{m}\varvec{u}\varvec{m} \varvec{s}\varvec{c}\varvec{o}\varvec{r}\varvec{e}}\varvec{*} 100\varvec{\%}$$

The level of therapeutic communication was done by using data-driven classification (tertiary classification by rank order); the lower tertiary represents the low level, the middle tertiary represents moderate level, and the upper tertiary indicates the higher level of therapeutic communication.

**Phases of the development of a therapeutic relationship**: therapeutic relationship takes place when professionals, specifically educated to be nurses, engage with people who need health services. A well-known nurse theorist named Hildegard Peplau stated this in three phases such as, orientation, exploitation (working), and resolution (termination). **Orientation phase**: is a problem-defining phase that starts when the client meets nurse as a stranger and focus on establishing a therapeutic environment. **Working phase**: a phase in which the client’s problems are identified and solutions are explored, applied, and evaluated. **Resolution phase**: the nurse terminates the relationship when the mutually agreed goals are met, the patient is discharged or transferred or the rotation is finished [25, 26].

### Data quality control

All the tools were discussed with experts who have deep knowledge of the subject matter to ensure construct, content, and face validity. A pre-testing of the tool and training of data collectors and supervisors were conducted before actual data collection, and necessary modifications and amendments were done. The standard tool with high internal consistency (Cronbach’s alpha = 0.93) was used to collect the data. Besides, data were collected by using ODK to control possible inconsistencies.

### Data analysis

The collected data were downloaded from ODK aggregate, exported to SPSS version 25 for cleaning, and then imported to SAS version 9.4 for analysis. The descriptive analysis was done by computing proportions and summary statistics. A generalized linear model (GLM) was used to identify factors for the therapeutic communication. All the assumptions were checked. All variables with P < 0.25 were included in the final model to control all possible confounders. The goodness of fit was tested by the R-Squared (R^2^). Multi-collinearity test was carried out to see the correlation between independent variables by using collinearity statistics, and Variance inflation factor (VIF) > 10 and tolerance (T) < 0.1 were considered suggestive of the existence of multi-collinearity. Adjusted Beta (β) with 95%CI was estimated to identify factors. In this study P-value, < 0.05 was considered to declare a result as statistically significant. Finally, the results were summarized and presented by using simple frequencies, summary measures, tables, and figures.

## Results

### Socio-demographic and professional characteristics

In this study, 408 nurses have involved, with a response rate of 97.37%. The mean and standard deviation of the age were 34.72 ± 6.43 years old. Out of the study participants, 277 (67.9%) were females, 278(68.1%) were orthodox religious followers, and 285 (69.9%) were married. The shift schedule varies depending on the unit. Most units have rotating shifts, but some have fixed shifts. One hundred thirty-one (32.1%) respondents were 6 to 10 years of work experience as a nurse, and 209 (51.2%) earned monthly salaries of 8001–9500 Ethiopian birr (Table 1).

**Table 1:** Socio-demographic and professional characteristics of the nurses working in the public hospitals of Gamo zone, southern Ethiopia, 2022 *(n = 408)*

Variables	Frequency	Percentage (%)
**Sex**		
Male	131	32.1
Female	277	67.9
**Age of the respondent (in year)**		
25–29	105	25.7
30–34	110	27.0
35–39	105	25.7
≥ 40	88	21.6
**Marital status**		
Single	105	25.7
Married	285	69.9
Widowed	18	4.4
**Professional rank**		
Junior nurse	8	2.0
Senior nurse	400	98.0
**Unit/ward**		
Medical	226	55.4
Surgical	182	44.6
**Year of work experience in nursing**		
≤ 5	86	21.1
6–10	131	32.1
11–15	112	27.5
16–20	52	12.7
≥ 21	27	6.6
**Qualification**		
Diploma	137	33.6
BSc	271	66.4
**Salary per month (ETB)**		
5000–6500	88	21.6
6501–8000	111	27.2
8001–9500	209	51.2

### Nurse-related factors

The mean score of the negative attitude of the patient (4.35 ± 0.87), low salary (4.89 ± 0.31), and challenging nursing tasks (4.26 ± 0.44) was high compared with the other variables. The mean score of nurses’ inability to answer patients’ questions (1.90 ± 0.63) and insufficient knowledge of communication skills among nurses (1.90 ± 0.59) was the lowest (Table 2).

**Table 2:** Nurse-related factors affecting therapeutic communication among nurses in the public hospitals of Gamo zone, southern Ethiopia, 2022 *(n = 408)*

Characteristics	Mean ± SD
Being overworked	4.20 ± 0.49
Shortage of nurses	4.05 ± 0.85
The negative attitude of the patient	4.35 ± 0.87
Nurse’s unpleasant experiences	2.40 ± 1.01
Patients non-compliance to treatment	2.76 ± 1.01
Nurses’ burn-out (physical & mental tiredness)	4.00 ± 0.95
Lack of enough time	3.29 ± 1.15
Poor relationship with colleagues	2.50 ± 1.05
Nurses’ inability to answer patients’ questions	1.90 ± 0.63
Insufficient knowledge on communication skills	1.90 ± 0.59
Reluctance to communicate	2.03 ± 0.68
Lack of empathy from nurses	2.18 ± 0.79
Lack of communication skills	2.13 ± 0.69
Lack of interest	3.59 ± 1.09
Low salary	4.89 ± 0.31
Nursing shift work	4.05 ± 0.71
Lack of welfare facilities for nurses	2.80 ± 1.11
Patient contact with different nurses	3.94 ± 0.84
Challenging nursing tasks	4.26 ± 0.44

### Patient-related factors

**Fig. 1:**
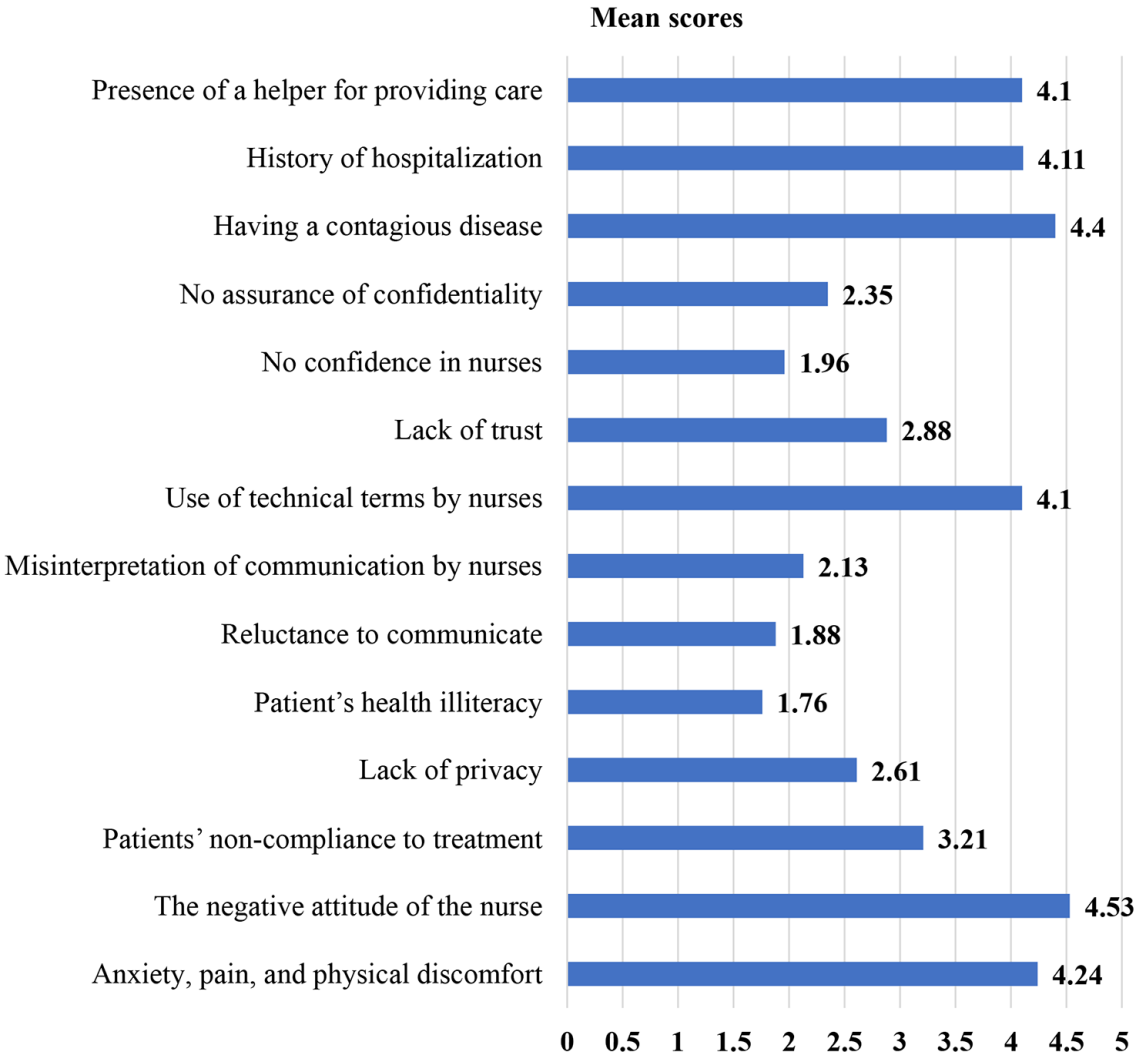
Mean scores of the patient-related factors affecting therapeutic communication among nurses in the public hospitals of Gamo zone, southern Ethiopia, 2022 *(n = 408)*

In this study, the mean score of anxiety, pain, physical discomfort, disease severity (4.24 ± 0.55), the negative attitude of the nurse (4.53 ± 0.54), and having a contagious disease (4.40 ± 0.61) was high compared with the other variables. Whereas the mean score of patient’s health illiteracy (1.76 ± 0.78), reluctance to communicate (1.88 ± 0.71), and low level of confidence among nurses (1.96 ± 0.65) were the lowest (Fig. 1).

### Environmental-related factors

Out of environmental-related factors, workload (4.26 ± 0.52), lack of nurses’ participation in decision-making (4.42 ± 0.83), and the feeling of injustice at the workplace (4.53 ± 0.50) had the highest mean score as compared to other characteristics. Nevertheless, lack of support by other staff (2.40 ± 0.97), poor job performance by other staff (2.17 ± 0.88), and lack of respect for opinions made by junior nursing staff (2.20 ± 1.03) was the lowest mean score (Table 3).

**Table 3:** Environmental-related factors affecting therapeutic communication among nurses in the public hospitals of Gamo zone, southern Ethiopia, 2022 *(n = 408)*

Characteristics	Mean ± SD
Workload	4.26 ± 0.52
Unsuitable environmental/Poor sanitation in patients’ rooms	2.99 ± 1.19
Stress-related issues	3.87 ± 0.77
Lack of support by other staff	2.40 ± 0.97
Staff shortage	3.71 ± 1.04
Poor communication between nurse &physicians	2.54 ± 0.96
The busy environment of the ward (noise and traffic)	3.49 ± 0.87
Nursing becoming task-oriented instead of patient-centered	4.19 ± 0.63
Poor job performance by other staff	2.17 ± 0.88
Lack of respect for opinions made by junior nursing staff	2.20 ± 1.03
The unfamiliar environment of the hospital for the patients	2.66 ± 1.03
Lack of welfare and medical facilities for patients	2.68 ± 1.04
Lack of continuing education in communication skills	2.73 ± 1.18
Lack of managerial appreciation from nurses	4.18 ± 1.25
Lack of educational background in communication skills	3.04 ± 1.27
Lack of nurses’ participation in decision-making	4.42 ± 0.83
The feeling of injustice at the workplace	4.53 ± 0.50

### Personal/social-related factors

**Fig. 2:**
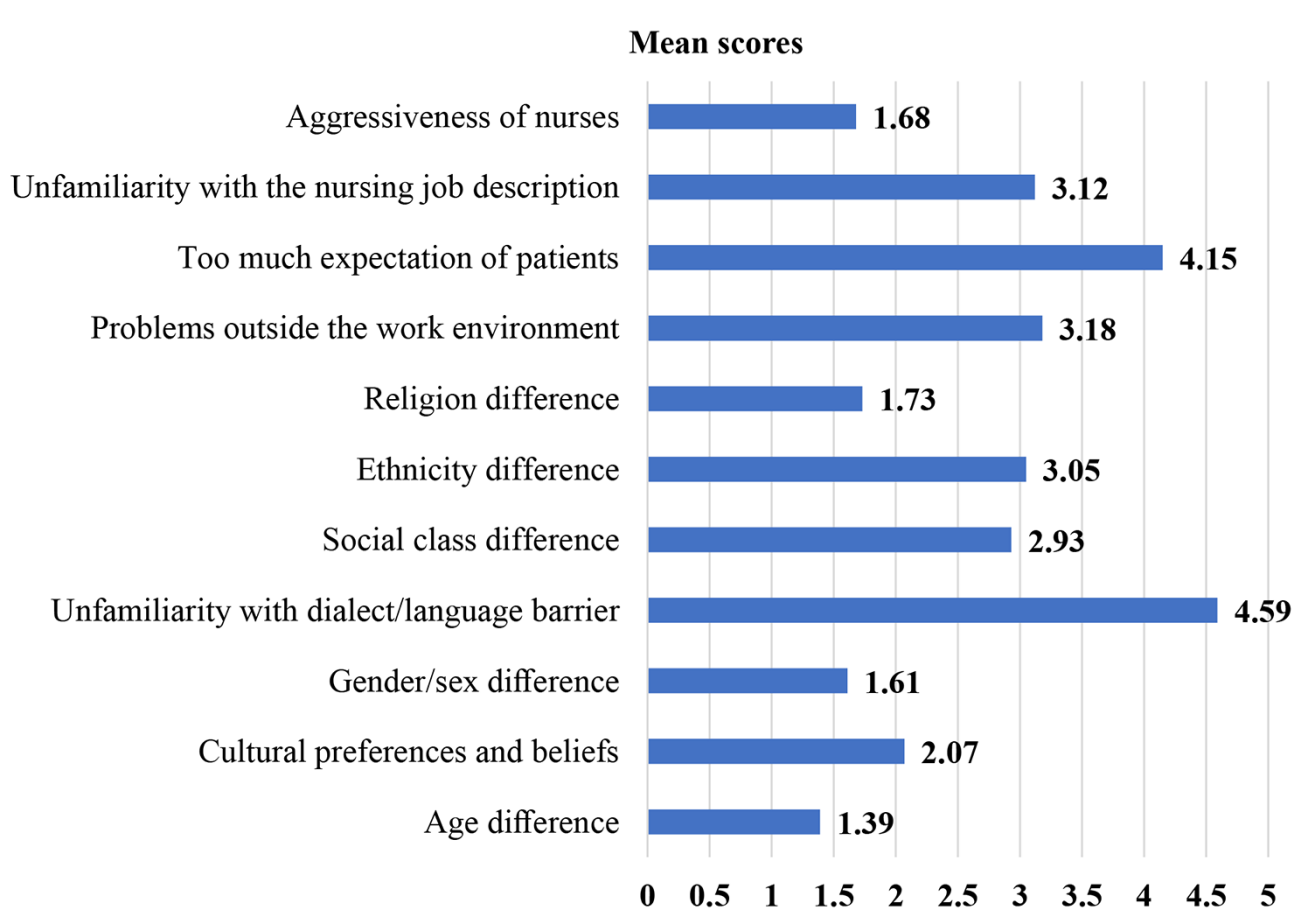
Mean scores of the personal/social -related factors affecting therapeutic communication among nurses in the public hospitals of Gamo zone, southern Ethiopia, 2022 *(n = 408)*

The mean score of unfamiliarity with dialect/language barrier (4.59 ± 0.80), problems outside the work environment (3.18 ± 0.97), and too much expectation of patients (4.15 ± 0.68) were higher from personal/social-related characteristics. Age difference (1.39 ± 0.69), gender/sex difference (1.61 ± 0.49), and aggressiveness of nurses (1.68 ± 0.86) showed the lowest mean score in this study (Fig. 2).

### Level and framework of therapeutic communication

**Fig. 3:**
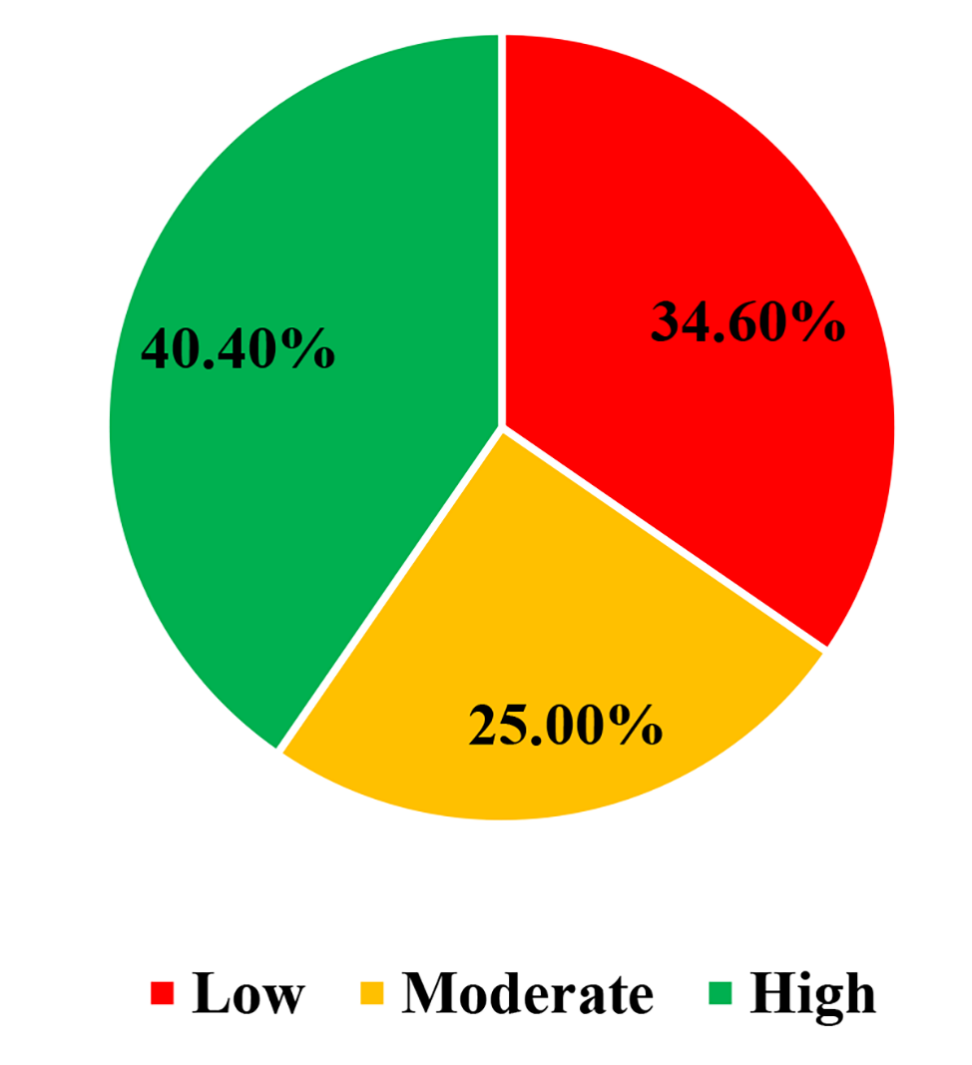
Level of therapeutic communication among nurses in the public hospitals of Gamo zone, southern Ethiopia, 2022 *(n = 408)*

**Fig. 4:**
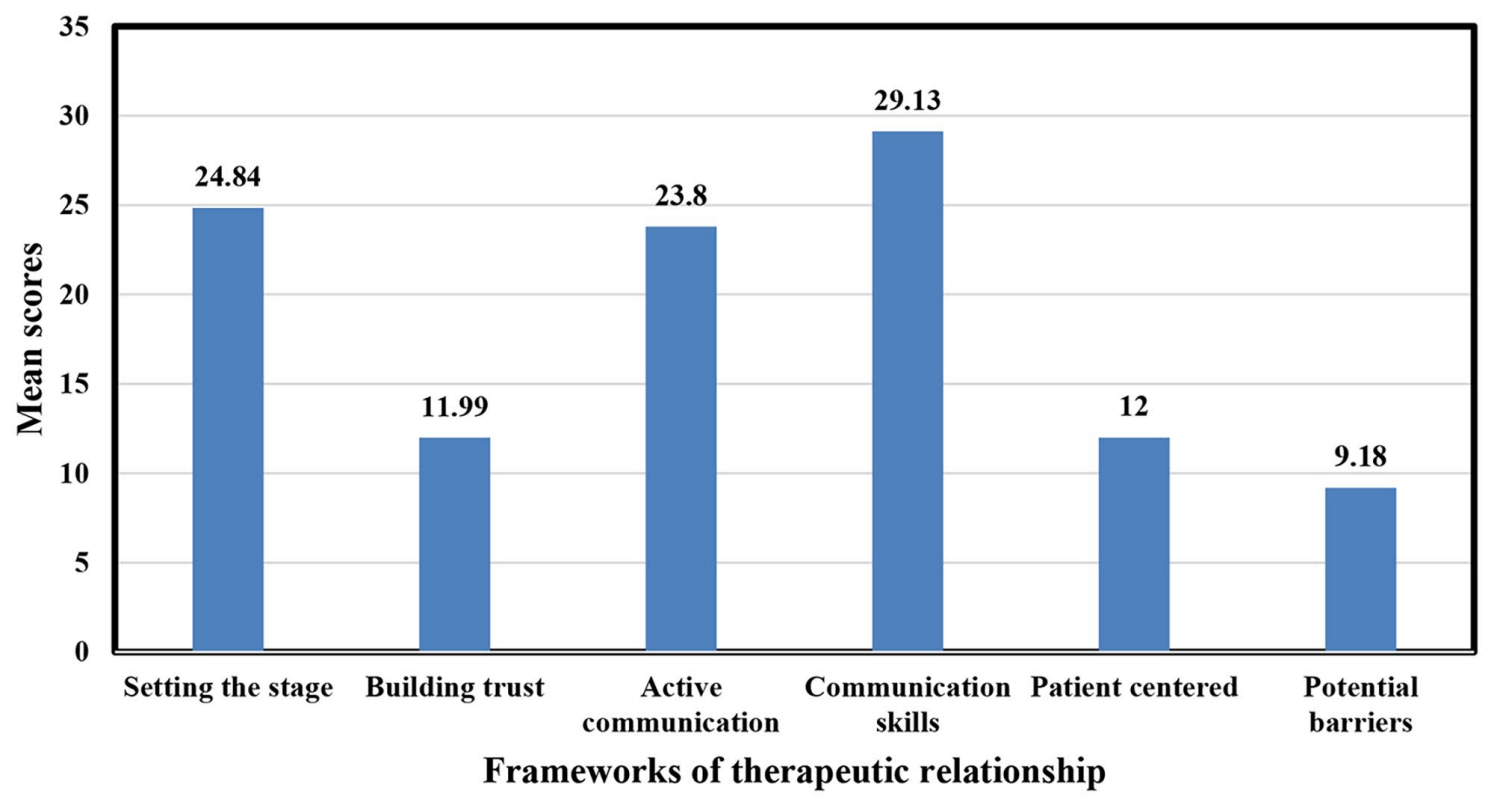
Mean scores of frameworks of therapeutic relationship in public hospitals of Gamo zone, southern Ethiopia, 2022 *(n = 408)*

The mean score of therapeutic communications of nurses was 110.94 (95%CI: 110.54, 111.34) and a standard deviation of 4.09. The %SM of therapeutic communication was 52.32%. In this study, 165 (40.4%) had a high level of therapeutic communication (Fig. 3). Out of the frameworks, of the therapeutic relationship, the mean score of setting the stage was 24.84 ± 1.21, and communication skills were 29.13 ± 1.58 (Fig. 4).

### Inter-relation between constructs of therapeutic communication and phases of Hildegard Peplau’s nursing theory of interpersonal relations

This study finding indicated that the constructs of therapeutic communications and Hildegard Peplau’s phases of the nurse-patient relationship were congruent. Consequently, power-sharing indicated the orientation phase with a %SM of 53.57%, trust and rapport building indicated the identification/exploitation (working) phase with a %SM of 57%, and empathy indicated the resolution (termination) phase with a %SM of 65%.

### Factors associated with therapeutic communication

After controlling the variance explained by all other variables in the generalized linear model, age, marital status, and qualification showed a significant and positive relationship with overall therapeutic communication. However, sex, working unit, nurse burnout, lack of empathy from nurses, challenging nursing tasks, lack of privacy, use of technical terms by nurses, confidence in nurses, stress, unfamiliarity with the nursing job description, shortage of nurses, knowledge, participation in decision making, and having contagious disease showed a significant and negative relationship with overall therapeutic communication.

A unit (one year) increase in age would increase the overall therapeutic communication score by 0.14 unit. Female nurses were 14% less likely to have therapeutic communication than male nurses. Widowed nurses were about seven times more likely to have therapeutic communication than single nurses in their marital status. Nurses working in the surgical ward were 82% less likely to have therapeutic communication than to nurses working in the medical ward.

The nurse with BSc degrees was 1.31 times more likely to have therapeutic communication than nurses with a diploma. A unit increase in the nurses’ burn-out (physical & mental tiredness) score would result in a 1.1 unit drop in the overall therapeutic communication score.

A unit increase in the lack of empathy from nurses would result in a 1.32 unit decrease in the overall therapeutic communication score. A unit increase in challenging nursing tasks would result in a 2.3 unit drop in the overall therapeutic communication score. A unit increase in the lack of privacy from the patient would result in a 1.62 unit decline in the overall therapeutic communication score. A unit increase in the reluctance of patients to communicate with nurses would result in a 0.25 unit decrease in the overall therapeutic communication score. A unit increase in use of technical terms by nurses would result in a 2.72 unit drop in the overall therapeutic communication score. A unit increase in the lack of confidence in nurses would result in a 1.52 unit decline in the overall therapeutic communication score.

A unit increase in the stress of nurses would result in a 1.62 unit decrease in the overall therapeutic communication score. When nurses’ unfamiliarity with their job description increased by one unit, the overall therapeutic communication score decreased by 0.94 units. A unit increase in the shortage of nurses would result in a 3.39 unit decrease in the overall therapeutic communication score. A unit increase in knowledge insufficiency in communication skills would result in a 3.36 unit drop in the overall therapeutic communication score.

A unit increase in the lack of nurses’ participation in decision-making would result in a 1.39 unit decline in the overall therapeutic communication score. A unit increase in patients with a contagious disease would result in a 1.44 unit decrease in the overall therapeutic communication score (Table 4).

**Table 4:** Regression coefficient of the factors associated with therapeutic communication among nurses working in public hospitals of Gamo zone, southern Ethiopia, 2022 *(n = 408)*

Variables	β estimate with 95%CI	P-value
Age	0.14(0.07,0.21)*	< 0.001
Sex (female)	-0.86(-1.71,-0.01)*	0.048
Marital status		
Married	0.01(-0.98,1.03)	0.96
Widowed	7.02(4.71,9.32)*	< 0.001
Ward (surgical)	-4.18(-5.02,-3.35)*	< 0.001
Qualification (BSc)	1.31(0.22,2.40)*	0.02
Nurses’ burn-out (physical & mental tiredness)	-1.10(-2.17,-0.04)*	0.04
Lack of empathy from nurses	-1.32(-2.53,-0.10)*	0.03
Challenging nursing tasks	-2.30(-3.48,-1.13)*	< 0.001
Lack of privacy	-1.62(-2.37,-0.88)*	< 0.001
Patient’s health illiteracy	0.46(-0.44,1.37)	0.32
Reluctance of patient to communicate	-0.25(-1.41,0.91)	0.68
Use of technical terms by nurses	-2.72(-4.18,-1.25)*	< 0.001
No confidence in nurses	-1.52(-2.77,-0.27)*	0.02
Poor sanitation in patients’ rooms	-0.69(-1.47,0.09)	0.08
Stress-related issues	-1.62(-2.72,-0.53)*	0.004
Age difference between nurses and patient	-1.06(-2.33,0.20)	0.09
Cultural preferences and beliefs	-0.68(-1.66,0.29)	0.17
Religion difference	0.76(-0.23,1.75)	0.13
Unfamiliarity with the nursing job description	-0.94(-1.73,-0.14)*	0.02
Shortage of nurses	-3.39(-4.78,-1.99)*	< 0.001
Insufficient knowledge in communication skills	-3.36(-4.59,-2.14)*	< 0.001
Lack of managerial appreciation from nurses	-0.59(-1.75,0.58)	0.33
Lack of nurses’ participation in decision-making	-1.39(-2.36,-0.42)*	0.005
Lack of communication skills	-0.58(-2.07,0.91)	0.45
Having a contagious disease	-1.44(-2.12,-0.77)*	< 0.001

*Significant at P < 0.05

## Discussion

Therapeutic communication between nurse and patient is a pillar in quality nursing care and improves patient outcomes. Ineffective nurse-patient communication adversely affects patient safety and patient satisfaction [27]. In Ethiopia, there is a gap in therapeutic communication between nurses and patients. These highly limit patient-centered care and patient satisfaction, and people may not trust the service delivery, putting patient outcomes under caution. Limited studies were conducted, and those studies identified a few factors. Therefore, this study aimed to estimate the level of therapeutic communication and factors affected by integrating Peplau’s nursing theory of interpersonal relations in the study setting.

This study indicated the equivalence of the constructs of therapeutic communication and phases of Peplau’s nursing theory of interpersonal relations. It also disclosed the applicability of Peplau’s nursing theory or model in our healthcare setting. A standardized percentage of the maximum scale of each phase of therapeutic communication in this study was optimum. The findings of this study showed slightly more than one-third of the study participants had a low level of therapeutic communication, and one-fourth of them scored a moderate level of therapeutic communication. This finding was in line with the other studies conducted in Ethiopia [28–30], Greece [31], and scoping review conducted in sub-Saharan Africa [32]. These may be due to the inadequacy of communication skills in teaching and experience in nursing. These suggest the need for advances in communication competence among nurses, nurse-to-patient communication policy review, and identification of possible barriers. However, this finding was inconsistent with the studies conducted in Brazil (25%) [33], Iran (14.3%) [34], Ethiopia (61.4%) [18], and Korea (75%) [35]. The inconsistency might be due to differences in workload, patient flow, nurses’ communication skills and experiences, nurse-to-patient interaction (lack of devotion to communicating), and level of patient engagement. The other possible reasons are also organizational factors affecting nurse-to-patient communication and the level of working environment conduciveness.

The findings of this study showed socio-demographic and professional characteristics such as age, sex, marital status, working unit, and nurses’ qualification were significantly associated with therapeutic communication. Burnout, knowledge, lack of empathy, and challenging nursing tasks were identified as nurse-related, and lack of privacy, use of technical terms, no confidence in nurses, and having a contagious disease from patient-related were factors that affect therapeutic communication. Likewise, stress, staff shortage, and lack of nurses’ participation in decision-making from environmental-related, and unfamiliarity with the nursing job description from personal-related were identified as factors affecting overall therapeutic communication. This finding was consistent with studies conducted in Ghana, Rwanda, Malawi, Mali, Nigeria, Botswana, Iran, and Ethiopia. Those studies identified age, cultural and religious beliefs, misconceptions, pain, language barriers, inadequate nurses, lack of communication skills, negative attitudes from patients, family interference, perceived patient and caregivers’ views, stress, and work overload as factors affecting the level of therapeutic communication [18, 21, 28–30, 36–43]. These are also identified and supported by scoping review conducted in sub-Saharan African counties [32]. The similarity might be due to common factors affecting therapeutic communication among nurses working in low socioeconomic countries. It is affected by multiple personal, organizational, and policy-related factors. Those are nursing workforce shortage which directly and indirectly causes work overload, minimal patient-to-nurse interaction, low level of patient engagement in care decisions, and low job satisfaction among nurses. Moreover, low level of therapeutic interactions between nurses and patients and the complexity of the working environment due to poor infrastructure and low level of nursing care quality in developing countries.

Therapeutic communication is one of the pillars of helping a client and opens the way to identifying different conditions of the patient. As such, the health care professionals in the health care system, typically nurses’ should give focus and take responsibility. Without effective communication, the patient cannot be satisfied, and the outcome is compromised. This study is theoretical applicability in the healthcare setting. It indicated that therapeutic communication was low and identified the main factors that interfere with effective communication. Our health care system is not advanced related to patient handling, and evidence reported most patients are not satisfied with the services given to them. The main contributing factor to patient satisfaction is effective communication with healthcare providers. If there is ineffective communication, patients do not trust them, and even they do not disclose some situations that may need immediate intervention. These findings also supplement by a pocket of studies and taken as tangible evidence. Therefore, concerned bodies should give attention and those identified factors that need intervention. In addition, stakeholders should also put their fingerprints on it, and other scholars may go further to show clear strategies for policymakers and program planners.

The main strength of this study is that it is integrated with the nursing model and shows a direction for the clinical applicability of the theory. The limitations are this study was subjected to social desirability bias as the participants may deviate from their response to the standard or the most acceptable conditions. The other drawback is that it is measured only from the perspective of nurses.

## Conclusions

The therapeutic communication between nurses and patients in this study is low. Socio-demographic and professional characteristics, burnout, knowledge, lack of empathy and challenging tasks, lack of privacy, use of technical terms, and no confidence in nurses’, are identified as factors that affect therapeutic communication. Likewise, having a contagious disease, stress, staff shortage, lack of nurses’ participation in decision-making, and unfamiliarity with the nursing job description are also identified. Even, this study complements the previous findings, the use of the theoretical concept of Peplau makes it unique and adds value to existing knowledge. This study is relevant to improve the quality of services in the health care system and used as input to designing appropriate strategies. The findings of this study are original and assessed by using a validated and reliable tool. Therefore, giving opportunities for nurses to improve their qualifications, a special attention to nurses working in stressful areas, sharing the burden of nurses, involving nurses and patients in decision-making, and motivating and creating a positive working environment is vital to improving therapeutic communication.

### Electronic supplementary material

Below is the link to the electronic supplementary material.

Supplementary Material 1. Additional file 1: English version questionnaire (pdf)

## Data Availability

The datasets generated and/or analyzed during the current study are not publicly available due to anonymity issues but are available from the corresponding author at reasonable request.

## List of abbreviations

GITCS: Global Inter-professional Therapeutic Communication Scale
GLM: Generalized Linear Model
IRB: Institutional Research Ethics Review Board
ODK: Open Data Kit
%SM: Standardized Percentage of the Maximum Scale
and WMA: World Medical Association
