# Supplementary material for: Therapeutic communication and its associated factors among nurses working in public hospitals of Gamo zone, southern Ethiopia: application of Hildegard Peplau’s nursing theory of interpersonal relations
Source: BMC Nurs. 2023 Oct 13;22:381. doi: 10.1186/s12912-023-01526-z (PMC10571273; doi:10.1186/s12912-023-01526-z)
Supplement: Supplementary file 1 — Supplementary Material 1. Additional file 1: English version questionnaire (pdf) [file 12912_2023_1526_MOESM1_ESM.docx]

English Version Questionnaire

Questionnaire developed to assess the status of therapeutic communication and its associated factors in public hospitals of Gamo zone, southern Ethiopia, 2022

| **SNo** | **Questions** | **Response** | **Skip** |
| --- | --- | --- | --- |
| **Part I: Socio-demographic characteristics of nurses** | | | |
| 101 | Sex | 1. Male 2. Female |  |
| 102 | Age | **_________(**in year) |  |
| 103 | Religion | 1. Orthodox 2. Protestant 3. Muslim 4. Catholic 5. Other_____________ |  |
| 104 | Marital status | 1. Single 2. Married 3. Divorced 4. Widowed 5. Separated due to work |  |
| 105 | Rank | 1. Junior nurse 2. Senior Nurse |  |
| 106 | Unit/ward | 1. Medical 2. Surgical |  |
| 107 | Year of work experience in nursing | __________( in year) |  |
| 108 | Qualification | 1. Diploma 2. BSc |  |
| 109 | Salary per month | ___________(in ETB) |  |

| **Part II: Nurses related factors: 5 points Likert scale** | | | | | | | |
| --- | --- | --- | --- | --- | --- | --- | --- |
| **SNo** | **Characteristics** | **SD** | **D** | **DN** | **A** | **SA** | **Remark** |
| 201 | Being overworked (workload on nurses) |  |  |  |  |  |  |
| 202 | Shortage of nurses |  |  |  |  |  |  |
| 203 | The negative attitude of the patient |  |  |  |  |  |  |
| 204 | Nurse’s unpleasant experiences |  |  |  |  |  |  |
| 205 | Patients non-compliance to treatment |  |  |  |  |  |  |
| 206 | Nurses’ burn-out (physical & mental tiredness) |  |  |  |  |  |  |
| 207 | Lack of enough time |  |  |  |  |  |  |
| 208 | Poor relationship with colleagues |  |  |  |  |  |  |
| 209 | Nurses’ inability to answer patients’ questions |  |  |  |  |  |  |
| 210 | Insufficient knowledge on communication skills |  |  |  |  |  |  |
| 211 | Reluctance to communicate |  |  |  |  |  |  |
| 212 | Lack of empathy from nurses |  |  |  |  |  |  |
| 213 | Lack of communication skills |  |  |  |  |  |  |
| 214 | Lack of interest |  |  |  |  |  |  |
| 215 | Low salary |  |  |  |  |  |  |
| 216 | Nursing shift work |  |  |  |  |  |  |
| 217 | Lack of welfare facilities for nurses |  |  |  |  |  |  |
| 218 | Patient contact with different nurses |  |  |  |  |  |  |
| 219 | Challenging nursing tasks |  |  |  |  |  |  |

***Remark: SD: Strongly Disagree, D: Disagree, DN: Don’t know, A: Agree, and SA: Strongly Agree***

| **Part III: Patient-related factors: 5 points Likert scale** | | | | | | | |
| --- | --- | --- | --- | --- | --- | --- | --- |
| **SNo** | **Characteristics** | **SD** | **D** | **DN** | **A** | **SA** | **Remark** |
| 301 | Anxiety, pain, and physical discomfort or disease severity |  |  |  |  |  |  |
| 302 | The negative attitude of the nurse |  |  |  |  |  |  |
| 303 | Patients’ non-compliance to treatment |  |  |  |  |  |  |
| 304 | Lack of privacy |  |  |  |  |  |  |
| 305 | Patient’s health illiteracy |  |  |  |  |  |  |
| 306 | Reluctance to communicate |  |  |  |  |  |  |
| 307 | Misinterpretation of communication by nurses |  |  |  |  |  |  |
| 308 | Use of technical terms by nurses |  |  |  |  |  |  |
| 309 | Lack of trust |  |  |  |  |  |  |
| 310 | No confidence in nurses |  |  |  |  |  |  |
| 311 | No assurance of confidentiality |  |  |  |  |  |  |
| 312 | Having a contagious disease |  |  |  |  |  |  |
| 313 | History of hospitalization |  |  |  |  |  |  |
| 314 | Presence of a helper for providing care |  |  |  |  |  |  |

***Remark: SD: Strongly Disagree, D: Disagree, DN: Don’t know, A: Agree, and SA: Strongly Agree***

| **Part IV: Environmental related factors : 5 points Likert scale** | | | | | | | |
| --- | --- | --- | --- | --- | --- | --- | --- |
| **SNo** | **Characteristics** | **SD** | **D** | **DN** | **A** | **SA** | **Remark** |
| 401 | Workload |  |  |  |  |  |  |
| 402 | Unsuitable environmental/Poor sanitation in patients’ rooms |  |  |  |  |  |  |
| 403 | Stress-related issues |  |  |  |  |  |  |
| 404 | Lack of support by other staﬀ |  |  |  |  |  |  |
| 405 | Staﬀ shortage |  |  |  |  |  |  |
| 406 | Poor communication between nurse &physicians |  |  |  |  |  |  |
| 407 | The busy environment of the ward (noise and traffic) |  |  |  |  |  |  |
| 408 | Nursing becoming task-oriented instead of patient-centered |  |  |  |  |  |  |
| 409 | Poor job performance by other staﬀ |  |  |  |  |  |  |
| 410 | Lack of respect for opinions made by junior nursing staﬀ |  |  |  |  |  |  |
| 411 | The unfamiliar environment of the hospital for the patients |  |  |  |  |  |  |
| 412 | Lack of welfare and medical facilities for patients |  |  |  |  |  |  |
| 413 | Lack of continuing education in communication skills |  |  |  |  |  |  |
| 414 | Lack of managerial appreciation from nurses |  |  |  |  |  |  |
| 415 | Lack of educational background in communication skills |  |  |  |  |  |  |
| 416 | Lack of nurses’ participation in decision-making |  |  |  |  |  |  |
| 417 | The feeling of injustice at the workplace |  |  |  |  |  |  |

***Remark: SD: Strongly Disagree, D: Disagree, DN: Don’t know, A: Agree, and SA: Strongly Agree***

| **Part V: Personal/social related factors : 5 points Likert scale** | | | | | | | |
| --- | --- | --- | --- | --- | --- | --- | --- |
| **SNo** | **Characteristics** | **SD** | **D** | **DN** | **A** | **SA** | **Remark** |
| 501 | Age diﬀerence |  |  |  |  |  |  |
| 502 | Cultural preferences and beliefs |  |  |  |  |  |  |
| 503 | Gender/sex diﬀerence |  |  |  |  |  |  |
| 504 | Unfamiliarity with dialect/language barrier |  |  |  |  |  |  |
| 505 | Social class difference |  |  |  |  |  |  |
| 506 | Ethnicity difference |  |  |  |  |  |  |
| 507 | Religion difference |  |  |  |  |  |  |
| 508 | Problems outside the work environment |  |  |  |  |  |  |
| 509 | Too much expectation of patients |  |  |  |  |  |  |
| 510 | Unfamiliarity with the nursing job description |  |  |  |  |  |  |
| 511 | Aggressiveness of nurses |  |  |  |  |  |  |

***Remark: SD: Strongly Disagree, D: Disagree, DN: Don’t know, A: Agree, and SA: Strongly Agree***

| **Part VI: Therapeutic communications assessment tool (Global Interprofessional Therapeutic**  **Communication Scale): 5 points Likert scale** | | | | | | | | |
| --- | --- | --- | --- | --- | --- | --- | --- | --- |
| **Categories** | **SNo** | **Characteristics** | **1** | **2** | **3** | **4** | **5** | **NA** |
| Setting the stage | 601 | Provides a professional greeting given the context |  |  |  |  |  |  |
|  | 602 | Introduces self by name and title without prompting |  |  |  |  |  |  |
|  | 603 | Conducts the communication in a culturally safe manner |  |  |  |  |  |  |
|  | 604 | Purposefully explains mutually established goals for the visit |  |  |  |  |  |  |
|  | 605 | Demonstrates appropriate proximity to the patient or family according to the context |  |  |  |  |  |  |
|  | 606 | Where possible provide for privacy and minimal interruptions during the interaction |  |  |  |  |  |  |
| Building trust | 607 | Verbalizes interest in the patient and their perspective, encouraging rapport |  |  |  |  |  |  |
|  | 608 | Demonstrates knowledge about the patient's case or situation |  |  |  |  |  |  |
|  | 609 | Encourages feedback and input from the patient |  |  |  |  |  |  |
| Active communication | 610 | Provides accurate information to the patient at the level they understand |  |  |  |  |  |  |
|  | 611 | Verifies comprehension (patient understanding information) |  |  |  |  |  |  |
|  | 612 | Explains differently if necessary according to the patient’s feedback |  |  |  |  |  |  |
|  | 613 | Uses questions in a balanced way, avoiding patient’s passive participation (e.g. only responding to questions) |  |  |  |  |  |  |
|  | 614 | Offers patient opportunities to organize and express their thoughts about the messages |  |  |  |  |  |  |
|  | 615 | Listens attentively and answers questions |  |  |  |  |  |  |
| Communication skills | 616 | Recognizes and responds to patient's non- verbal reactions |  |  |  |  |  |  |
|  | 617 | Speaks in an appropriate tone and volume given the situation |  |  |  |  |  |  |
|  | 618 | Sits or remains level with the patient when possible given the context/situation |  |  |  |  |  |  |
|  | 619 | Maintains contact appropriate to the culture when talking with the patient and/or family (e.g. eye contact, distance, spatial approximation) |  |  |  |  |  |  |
|  | 620 | Describes what they are going to do before doing it |  |  |  |  |  |  |
|  | 621 | Asks permission to touch before doing anything to the patient (e.g. blood pressure, dressing, palpation) |  |  |  |  |  |  |
|  | 622 | Touches the patient in a culturally respectful manner |  |  |  |  |  |  |
| Patient centered | 623 | Seeks input from the patient regarding their feelings and goals |  |  |  |  |  |  |
|  | 624 | Provides balanced time on psychosocial and clinical aspects of patient care depending on the context |  |  |  |  |  |  |
|  | 625 | Identifies potential conflict and finds opportunities to gather information to minimize or manage it |  |  |  |  |  |  |
| Potential barriers | 626 | Gives advice rather than explain options and alternatives* |  |  |  |  |  |  |
|  | 627 | Gives unsupported (false) reassurance* |  |  |  |  |  |  |
|  | 628 | Infers falsely, jumps to conclusions related to the client’s behaviors* |  |  |  |  |  |  |

***Remark: 1: Never, 2: Rarely, 3: Sometimes, 4: Usually, 5: Always, NA: Not applicable, and*** ******Reverse items***

***Many Thanks!***
